# Neuromorphic computing facilitates deep brain-machine fusion for high-performance neuroprosthesis

**DOI:** 10.3389/fnins.2023.1153985

**Published:** 2023-05-12

**Authors:** Yu Qi, Jiajun Chen, Yueming Wang

**Affiliations:** ^1^Affiliated Mental Health Center & Hangzhou Seventh People’s Hospital, MOE Frontier Science Center for Brain Science and Brain-Machine Integration, Zhejiang University School of Medicine, Hangzhou, China; ^2^Qiushi Academy for Advanced Studies, Zhejiang University, Hangzhou, China

**Keywords:** brain-machine interface, brain-computer interface, neuromorphic model, brain-like computing, neuroprosthesis, brain-machine fusion

## Abstract

Brain-machine interfaces (BMI) have developed rapidly in recent years, but still face critical issues such as accuracy and stability. Ideally, a BMI system would be an implantable neuroprosthesis that would be tightly connected and integrated into the brain. However, the heterogeneity of brains and machines hinders deep fusion between the two. Neuromorphic computing models, which mimic the structure and mechanism of biological nervous systems, present a promising approach to developing high-performance neuroprosthesis. The biologically plausible property of neuromorphic models enables homogeneous information representation and computation in the form of discrete spikes between the brain and the machine, promoting deep brain-machine fusion and bringing new breakthroughs for high-performance and long-term usable BMI systems. Furthermore, neuromorphic models can be computed at ultra-low energy costs and thus are suitable for brain-implantable neuroprosthesis devices. The intersection of neuromorphic computing and BMI has great potential to lead the development of reliable, low-power implantable BMI devices and advance the development and application of BMI.

## 1. Introduction

Brain-machine interface (BMI) is a technology that enables direct interaction between the brain and external devices such as cursors, robotic arms, and prosthetic limbs, which has demonstrated great potential in various applications, including gaming, smart homes, and neural or motor rehabilitation (Hochberg et al., 2012).

Most recently, intracortical brain-machine interfaces (iBMI), which decode information from single-neuron-level neural signals, have seen rapid progress and enabled new forms of neuroprosthesis, such as brain-to-handwriting (Willett et al., 2021), BMI-based speech synthesis (Moses et al., 2021), and implantable neural therapies for epilepsy (Berényi et al., 2012) and depression (Scangos et al., 2021). The emergence of BMI technology companies, represented by Neuralink, has sparked a wave of rapid development of brain-implantable hardware and devices, boosting the clinical application of BMIs.

## 2. Challenges for high-performance BMIs

Ideally, an iBMI system would take the form of brain-implantable neuroprosthesis and would work collaboratively with the brain, like an extension of the brain (Wu et al., 2016). The brain and the iBMI-based neuroprosthesis should be closely connected and integrated, with both sides adapting to, learning with, and compensating for each other as one. However, such a deep connection is difficult to achieve, given the fundamental difference between the brain and the machine. Specifically, from the side of the biological brain, information is encoded in spike trains of neurons. While from the side of computing machines, the basic unit for computation is vectors in real values. The gap between representation and computing lays a barrier to deep fusion between brain and machine, degrading the performance of iBMI systems. Lacking the deep connection between the brain and the machine, the existing iBMI systems still face critical challenges that have seriously hindered clinical application, including:

### 2.1. Degree of freedom and accuracy

Most motor iBMIs can only control 2–3 degrees of freedom at the same time, typical applications include 2D cursors and 3D robotic arms. The accuracy of the online control process is around 60–90% with full brain control with a path efficiency of 0.4–0.8 (Collinger et al., 2013; Wodlinger et al., 2014), which still cannot meet the clinical use requirements.

### 2.2. Adaptation

Most existing BMI systems lack the ability to adapt over time and exhibit limited cross-day or long-term performance (Qi et al., 2019; Degenhart et al., 2020). Since brain signals change dynamically over time, a BMI system usually has to be recalibrated every day to maintain its performance, which seriously affects the user experience (Brandman et al., 2018).

### 2.3. Low-cost computing

In particular, brain signals are high-throughput data, and neural decoding approaches are commonly energy-intensive, leading to issues such as low battery life. Thus, most existing brain-implantable devices only contain a limited number of channels (usually below 50 recording channels) (Rosenthal and Reynolds, 2019; Shupe et al., 2021). Especially, for brain-implantable devices, existing wireless devices usually cannot continuously work for more than 1–2 days (Shaikh et al., 2019).

## 3. Neuromorphic computing facilitates deep brain-machine fusion

**FIGURE 1:**
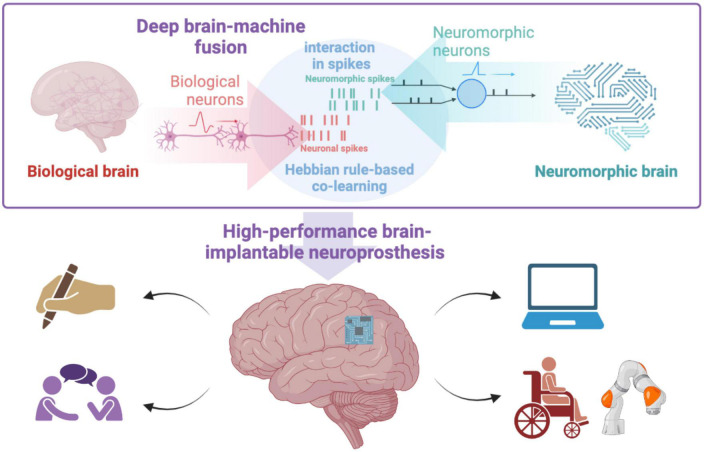
Neuromorphic computing facilitates deep brain-machine fusion.

Neuromorphic computing models, which mimic the structure and mechanism of biological neural circuits, provide a promising new option for building high-performance neuroprosthesis (Figure 1).

Neuromorphic computing technologies, such as spiking neural networks (SNNs), simulate neuron models of the brain and compute in the form of discrete spikes (Maass, 1997). Computational neuron models, such as the Hodgkin–Huxley model, spike response model, and leaky integrate-and-fire (LIF), mimicking the behavior of biological neurons, are the basic unit for information representation and computation. And the learning process is based on discrete spikes generated by the neuromorphic neurons, following the Hebbian rules and spike timing-dependent plasticity (STDP) rule that resemble biological nervous systems. Additionally, supervised algorithms like tempotron and resume (Gütig and Sompolinsky, 2006), which are derived from artificial neural network technologies, can also be utilized in the learning process.

Another advantage of neuromorphic computing is the ability of ultra-low-cost computing. The spike-based computing is an event-driven asynchronous process, which greatly saves computing energy consumption and realizes ultra-low power consumption computing deployed on neuromorphic chips. Taking partial integro-differential equations solving task as an example, the neuromorphic computing systems simulate the brain’s neural processes, the neuromorphic computing chip TrueNorth (Merolla et al., 2014) demonstrates a much lower power consumption (10^–3^ to 10^–1^ W) than commodity server-class computing chips (such as the Intel Xeon E5-2662, which consumes around 10^2^ W) (Smith et al., 2022), while still achieving comparable performance.

These features make the neuromorphic computing model a suitable option for developing such high-performance neuroprosthesis.

### 3.1. Providing a deep and precise connection between brain and machine

With the natural biological plausibility, neuromorphic models enable homogeneous information representation and computation between brain and machine, by direct information transfer in the form of spike trains, which can potentially enclose the connection between both sides. Traditionally, neuronal spike trains are transformed into continuous values in temporal bins to be fed into decoders (Hochberg et al., 2012; Willett et al., 2021), where the precise timing and spike order between neurons are inevitably lost. The direct spike-based interaction between brain and machine enables more precise information transfer, thus can boost the accuracy and stability of BMI systems.

### 3.2. Facilitating brain-machine co-adaptation

With the Hebbian learning rule that is shared between biological neurons and neuromorphic neurons, BMI systems can learn and develop adaptively with the brain in an online process, which is able to bring new breakthroughs for long-term BMI systems. Besides, neuromorphic models are also expected to overcome the issue of “catastrophic forgetting,” which is prevalent in current machine learning models (Imam and Cleland, 2020). They thus are able to perform continuous learning, and facilitate long-term and stable BMIs.

### 3.3. Enabling fully-implantable BMI devices

With the assistance of neuromorphic chips, neuromorphic models can compute with ultra-low energy cost (Basu et al., 2018), providing an ideal solution for wireless fully brain-implantable neuroprosthesis devices (Shaikh et al., 2019).

Currently, although there are only a few studies on the intersection of neuromorphic computing and BMI, they demonstrate the potential advantages of neuromorphic-model-based neural decoding. Imam and Cleland (2020) proposed a neuromorphic olfactory circuit for online learning of odor recognition and demonstrated the superiority of neuromorphic models in online one-shot learning and continuous learning. Li et al. (2019) proposed a “bioelectronic nose” using SNN decoder to decode odor information from neural activities recorded from the olfactory bulb of rats, demonstrating that neuromorphic models have improved performance and sensitivity (quicker response) compared to traditional machine learning approaches. Kasabov (2014) proposed a special neuromorphic model called NeuCube, which has demonstrated superior performance in brain signal processing tasks. Dethier et al. (2013) implemented a Kalman filter with spike computing and constructed a real-time cursor control BMI system, and found that a neuromorphic network with 2,000 neurons can achieve a success rate of over 94%, and the performance is stably maintained for at least 1 h in a pinball task. These studies demonstrated the advantages of neuromorphic model-based BMIs to some extent, while the deep fusion between the brain and machine, and the close intersection between neuromorphic computing and BMI is to be studied. Especially, with the advantages of neuromorphic computing models, the performance of BMI can be improved in both accuracy and stability, and BMI devices can hopefully meet the requirements of being small, energy-efficient, and fully brain-implantable, which could greatly benefit the clinical use and commercialization of BMIs.

## 4. Discussion

The field of BMI is currently in a period of rapid development. Neuromorphic computing, with its advantages of biological plausibility, continuous learning, and ultra-low energy consumption, perfectly aligns with the core challenges that BMI faces. The intersection of neuromorphic computing and BMI holds immense promise for the development of reliable and low-power implantable BMI devices and would significantly improve the long-term stability and usability of BMIs.

## Data availability statement

The original contributions presented in this study are included in the article/supplementary material, further inquiries can be directed to the corresponding authors.

## Author contributions

YQ proposed the perspective and wrote the manuscript. JC contributed to the manuscript writing. YW provided funding and contributed to the paper manuscript. All authors contributed to the article and approved the submitted version.
